# Analysis of risk factors for kinesiophobia after coronary artery bypass grafting and construction of a nomogram prediction model

**DOI:** 10.3389/fcvm.2026.1742685

**Published:** 2026-10-26

**Authors:** Lancai Guo, Jing Li, Wenjuan Ye, Xue Wei, Xia Wu, Xiaocan Wang

**Affiliations:** 1 Cardiac and Vascular Surgery ICU, The First Affiliated Hospital of Anhui Medical University, Hefei, Anhui, China; 2 Department of Cardiovascular Medicine, The First Affiliated Hospital of Anhui Medical University, Hefei, Anhui, China

**Keywords:** CABG, coronary artery bypass grafting, kinesiophobia, nomogram prediction model, risk factors, cardiac rehabilitation

## Abstract

**Background:**

Kinesiophobia, or the irrational fear of movement, is a significant barrier to effective cardiac rehabilitation and recovery in patients after coronary artery bypass grafting (CABG). This study sought to identify the risk factors for kinesiophobia in post-CABG patients and to develop and validate a nomogram prediction model to estimate individual risk.

**Methods:**

A retrospective case-control study was conducted on 138 patients who underwent CABG in the Cardiovascular ICU between February 2023 and November 2024. Using the Tampa Scale for Kinesiophobia-Heart (TSK-Heart), patients were categorized into a kinesiophobia group (TSK-Heart >37, *n* = 95) and a non-kinesiophobia group (*n* = 43). Risk Factors were identified with multivariable logistic regression. The resulting coefficients furnished a nomogram for individual risk estimation. Calibration was evaluated with the Hosmer-Lemeshow test and a bootstrap-corrected calibration plot; discrimination was quantified by the receiver operating characteristic (ROC) curve and calibration curve.

**Results:**

The incidence of kinesiophobia in this cohort was 68.84%. The logistic regression analysis identified six risk factors: advanced age (*OR* = 1.127, *P* = 0.003), lower educational level (*OR* = 0.246, *P* = 0.001), higher degree of disability in activities of daily living (ADL) (*OR* = 3.880, *P* = 0.007), lower self-efficacy in cardiac rehabilitation exercise (*OR* = 0.898, *P* = 0.024), pain catastrophizing (*OR* = 4.513, *P* = 0.023), and fear of falling (*OR* = 1.212, *P* = 0.004). The constructed nomogram demonstrated excellent discrimination, with an AUC of 0.939 (95% *CI*: 0.898–0.981), and high sensitivity (93.7%) and specificity (83.7%). Calibration was confirmed by the Hosmer-Lemeshow test (*χ*^2^ = 5.97, *P* = 0.65) and a bootstrap-corrected calibration plot showing close agreement between predicted and observed probabilities.

**Conclusions:**

Advanced age, low educational attainment, ADL dependency, poor cardiac rehabilitation self-efficacy, pain catastrophizing, and fear of falling were significant risk factors for kinesiophobia after CABG. The developed nomogram was a reliable and accurate tool for early identification of high-risk patients, enabling clinicians to implement individualized preventative strategies and optimize postoperative recovery.

## 1 Introduction

Coronary heart disease (CHD) remains a leading cause of morbidity and mortality worldwide (1). For patients with complex multivessel or left main coronary artery disease, coronary artery bypass grafting (CABG) is a highly effective revascularization strategy that significantly improves survival and quality of life (2, 3) Postoperative cardiac rehabilitation, with exercise training as its cornerstone, is universally recognized as critical for enhancing cardiopulmonary function, improving exercise capacity, and reducing long-term mortality and rehospitalization rates (4).

However, a substantial psychological barrier often impedes participation in rehabilitation: kinesiophobia, an excessive and irrational fear of movement and physical activity due to a perceived vulnerability to injury or pain (5). Following a major surgical intervention like CABG, patients may fear that physical exertion could precipitate adverse events such as sternal wound dehiscence, graft failure, or acute cardiac events. This fear-avoidance behavior fosters physical deconditioning, prolongs recovery, and worsens long-term clinical outcomes. With a pooled prevalence of 40%–70% after CABG, kinesiophobia represents a clinically significant yet modifiable determinant of recovery (6).

Therefore, the early identification of patients at high risk for kinesiophobia is paramount for implementing targeted, preventative interventions. While the issue is gaining recognition, there is a scarcity of robust predictive tools tailored to the post-CABG population. Accordingly, we sought to elicit risk factors of post-CABG kinesiophobia and develop and internally validate a clinically intuitive nomogram that quantifies individual risk to facilitate evidence-based, personalized care.

## 2 Methods

### 2.1 Study design and participants

A retrospective case-control study was conducted involving patients who underwent CABG at the Department of Cardiovascular ICU, The First Affiliated Hospital of Anhui Medical University from February 2023 to November 2024. A convenience sampling method was used. Inclusion criteria were: (1) confirmed diagnosis of CHD and indication for CABG; (2) age≥40 years; (3) voluntary participation and provision of informed consent; and (4) sufficient cognitive function to complete the questionnaires. Patients were excluded if they had (1) incomplete clinical data, (2) a history of psychiatric disorders, (3) previous CABG surgery, or (4) severe organic disease in other systems.

Based on prior study suggesting a kinesiophobia incidence of approximately 61% in cardiac surgery patients (7) a sample size of at least 115 participants was calculated to be necessary to achieve a statistical power of 95% with an alpha of 0.05 and a precision (e) of 0.1, after accounting for a 20% potential dropout rate (formula: *n* = *Z*^2^ × *P*(1－*P*)/e^2^. A total of 145 patients were initially enrolled, and 138 provided complete data, resulting in a valid response rate of 95.17%.

### 2.2 General information and questionnaire

Demographic information was collected, including age, sex, educational level, monthly family income, place of residence, primary caregiver(defined as the individual primarily responsible for the patient's daily care during the postoperative recovery period), and smoking and alcohol habits. Clinical data included body mass index (BMI), disease duration, left ventricular ejection fraction, number of comorbidities(defined as the total count of the following seven conditions: hypertension, diabetes mellitus, hyperlipidemia, COPD, chronic kidney disease, cerebrovascular disease, and peripheral vascular disease), number of grafts, duration of ICU stay, and duration of mechanical ventilation.

### 2.3 Tampa scale for kinesiophobia heart (TSK-heart)

The TSK-Heart was used to assess patients’ fear of movement and physical activity following cardiac events. This 17-item scale measures four dimensions: fear of movement, movement avoidance, danger perception, and functional impairment. The total score ranges from 17 to 68, with higher scores indicating a more severe level of kinesiophobia. A total score greater than 37 was used to classify a patient as having kinesiophobia. The Chinese version of the TSK-Heart was translated and culturally adapted using a rigorous forward-backward procedure, and its reliability and validity have been specifically established in Chinese patients with heart disease, demonstrating satisfactory internal consistency (Cronbach's *α* = 0.859) and construct validity (8).

### 2.4 Barthel index

The Barthel Index was used to evaluate a patient's independence in Activities of Daily Living (ADL). This 10-item scale has a total score ranging from 0 to 100, where higher scores reflect greater independence. A score ≤60 was defined as ADL disability and was further stratified into three levels: mild (51–60), moderate (41–50), and severe (≤40). The Chinese version of the Barthel Index has been well-validated in Chinese populations, with satisfactory reliability and predictive validity for functional outcomes (9).

### 2.5 Cardiac exercise self-efficacy instrument (CESEI)

The CESEI, a 16-item scale, was used to measure a patient's self-efficacy regarding postoperative rehabilitation exercises. Total scores range from 16 to 80, with higher scores indicating a greater level of self-efficacy. The psychometric properties of the Chinese version of the CESEI have been rigorously tested in Chinese patients with cardiovascular disease, demonstrating good internal consistency and test-retest reliability (10).

### 2.6 Pain catastrophizing scale (PCS)

The PCS is a 13-item scale used to assess a patient's level of pain catastrophizing. Scores range from 0 to 52, where higher scores reflect more severe catastrophizing sensations. A score ≥30 was used as the cutoff for significant pain catastrophizing. The Chinese version of the PCS (HK-PCS) has been formally validated in Chinese patients with chronic pain, and the cutoff of ≥30 has been confirmed as appropriate for identifying clinically relevant pain catastrophizing in this population (11).

### 2.7 Fear of falling questionnaire–revised (FFQ-R)

The FFQ-R, a 15-item scale, was utilized to measure the degree of a patient's fear of falling. Total scores range from 15 to 60, with higher scores indicating a more profound fear of falling. The reliability and validity of the Chinese version of the FFQ-R have been well documented in Chinese older adults, showing adequate internal consistency and structural validity (12).

All questionnaires were administered in a quiet setting by uniformly trained research personnel. The TSK-Heart was administered first, followed by the Barthel Index, CESEI, PCS, and FFQ-R in a fixed order, to minimize the potential influence of other questionnaires on the assessment of kinesiophobia. All assessments were conducted on postoperative day 3, with patients in a stable condition, and were completed within approximately 15 min during the morning hours to reduce circadian variability in mood and fatigue.

### 2.8 Statistical analyses

The characteristics of the participants were described using the mean ± standard deviation or frequency [percentage, *n*(%)]. Continuous variables were compared using Student's t-test (for normally distributed data) or the Mann–Whitney U test (for skewed data). Categorical variables were compared using the chi-square test or Fisher's exact test.

Variables that were statistically significant (*P* < 0.05) in the univariate analysis were entered into a multivariate logistic regression model using a backward stepwise method to identify independent predictors of kinesiophobia. The results are reported as odds ratios (*OR*) with 95% confidence intervals (*CI*). A *P*-value < 0.05 was considered statistically significant. The final model included six predictors with 95 kinesiophobia events, yielding an event-to-variable ratio of 15.8:1 (95 events ÷ 6 predictors). While this ratio exceeds the commonly accepted threshold of 10:1, the exceptionally high AUC (0.939) suggests potential overfitting. We addressed this by rigorous internal validation (1,000 bootstrap resamples).

The nomogram was constructed using the “rms” package in R software, the goodness of fit test was applied to the Hosmer-Lemeshow test, and the ROC curve and calibration curve were used to evaluate the model fitting. *P* < 0.05 was statistically significant. Statistical analysis was performed using SPSS version 23.0 (IBM Corp., Armonk, NY, USA) and R software version 4.1.2.

## 3 Results

### 3.1 Incidence of kinesiophobia

Among the 138 patients, 95 (68.84%) were classified into the kinesiophobia group. The mean TSK-Heart score for the entire cohort was 43.65 ± 7.90. The univariate analysis revealed that patients in the kinesiophobia group were significantly older, more likely to be female, had a lower educational level, a higher number of comorbidities, more severe ADL disability, longer postoperative mechanical ventilation time, lower CESEI scores, and higher PCS and FFQ-R scores (all *P* < 0.05). Detailed results are shown in Table 1.

**Table 1:** Univariate analysis of kinesiophobia in patients after CABG.

Variables	Non-kinesiophobia Group (*n* = 43)	Kinesiophobia Group (*n* = 95)	*χ*^2^*/Z/t* value	*P* value
Age (years)	58.19 ± 8.15	66.46 ± 7.92	5.634	<0.001
Sex [n(%)]			5.396	0.020
Male	36 (83.72)	61 (64.21)		
Female	7 (16.28)	34 (35.79)		
Education Level [*n*(%)]			4.621	<0.001
Primary school and below	9 (20.93)	48 (50.53)		
Junior high school	11 (25.58)	35 (36.84)		
High school and above	23 (53.49)	12 (12.63)		
Monthly Family Income [*n*(%)]			0.193	0.847
<4,000 yuan	13 (30.23)	29 (30.53)		
4,000～8,000 yuan	19 (44.19)	44 (46.32)		
>8,000 yuan	11 (25.58)	22 (23.16)		
Residence [*n*(%)]			0.553	0.758
Urban	12 (27.91)	21 (22.11)		
Township	14 (32.56)	34 (35.79)		
Rural	17 (39.53)	40 (42.11)		
Primary Caregiver [*n*(%)]			8.876	0.012
Children	26 (60.47)	34 (35.79)		
Spouse	12 (27.91)	52 (54.74)		
Caregiver and others	5 (11.63)	9 (9.47)		
Smoking History [*n*(%)]			0.115	0.908
None	18 (41.86)	38 (40.00)		
Occasionally	11 (25.58)	30 (31.58)		
Frequently	14 (32.56)	27 (28.42)		
Drinking History [*n*(%)]			0.465	0.642
None	15 (34.88)	36 (37.89)		
Occasionally	15 (34.88)	34 (35.79)		
Frequently	13 (30.23)	25 (26.32)		
BMI (kg/m^2^)	22.90 ± 2.34	22.60 ± 2.59	0.640	0.523
Disease Duration [*n*(%), year]			−0.036	0.972
＜1	22 (51.16)	52 (54.74)		
1–3	13 (30.23)	19 (20.00)		
>3	8 (18.61)	24 (25.26)		
Ejection Fraction [n(%)]			0.432	0.511
＜50%	16 (37.21)	41 (43.16)		
≥50%	27 (62.79)	54 (56.84)		
Comorbidities [*n*(%)]			2.098	0.036
≤1	8 (18.60)	5 (5.26)		
2	6 (13.95)	12 (12.63)		
≥3	29 (67.44)	78 (82.11)		
Number of Grafts [n(%)]			5.451	0.020
≤2	30 (69.77)	46 (48.42)		
>2	13 (30.23)	49 (51.58)		
ICU Stay Duration (hours)	26 (20.10,44.80)	25 (19.80,44.50)	0.480	0.631
Ventilator Use Duration (hours)	11 (8.00,14.00)	13.2 (9.20,18.00)	2.080	0.037
ADL Impairment Severity [n(%)]			4.402	<0.001
Mild	13 (30.23)	4 (4.21)		
Moderate	21 (48.84)	41 (43.16)		
Severe	9 (20.93)	50 (52.63)		
CESEI (scores)	54.00 ± 7.97	49.08 ± 5.68	4.133	<0.001
PCS [*n*(%)]			12.195	<0.001
<30	31 (72.09)	38 (40.00)		
≥ 30	12 (27.91)	57(60.00)		
FFQ-R(scores)	39.98 ± 4.76	43.62 ± 5.42	3.796	<0.001

### 3.2 Multivariate logistic regression analysis

The multivariate logistic regression analysis confirmed that six variables were independent predictors of kinesiophobia post-CABG: age (per 1-year increase: OR = 1.127, 95% CI: 1.042–1.219), educational level (per level increment: OR = 0.246, 95% CI: 0.105–0.576), ADL disability (moderate/severe vs. mild: OR = 3.880, 95% CI: 1.456–10.344), CESEI score (per 1-point increase: OR = 0.898, 95% CI: 0.819–0.986), PCS score (≥30 vs. < 30: OR = 4.513, 95% CI: 1.229–16.581), and FFQ-R score (per 1-point increase: OR = 1.212, 95% CI: 1.064–1.380). The detailed regression results are presented in Table 2 and Supplementary Table S1.

**Table 2:** Multivariate logistic regression analysis of kinesiophobia in post-CABG patients^a^.

Variables	*β*	Standard Error	Wald χ^2^ Value	*P* Value	*OR* Value	95%*CI*	
						Lower	Upper
Sex	0.695	0.778	0.797	0.372	2.003	0.436	9.209
Age	0.119	0.04	8.969	0.003	1.127	1.042	1.219
Education Level	−1.401	0.433	10.45	0.001	0.246	0.105	0.576
Caregiver (Children as reference)					1.000		
Spouse	0.356	0.671	0.282	0.596	1.428	0.383	5.321
Caregiver and others	0.018	1.004	0.000	0.986	1.018	0.142	7.281
Number of Comorbidities	0.764	0.499	2.343	0.126	2.148	0.807	5.715
Number of Grafts	0.938	0.632	2.202	0.138	2.555	0.74	8.822
Ventilator Use Duration	0.023	0.028	0.657	0.417	1.023	0.968	1.082
ADL Impairment Severity	1.356	0.500	7.348	0.007	3.88	1.456	10.344
CESEI	−0.107	0.047	5.093	0.024	0.898	0.819	0.986
PCS	1.507	0.664	5.153	0.023	4.513	1.229	16.581
FFQ-R	0.192	0.067	8.333	0.004	1.212	1.064	1.38

a Education level (ordinal variable).

### 3.3 Construction and validation of the nomogram

A nomogram for predicting the probability of kinesiophobia was developed based on the six independent predictors identified in the regression analysis (Figure 1). Each predictor level is assigned a point value, and the sum of these points corresponds to a specific predicted probability of developing kinesiophobia. The nomogram demonstrated excellent discriminative ability, with an AUC of 0.939 (95% *CI*: 0.898–0.981), a sensitivity of 93.7%, and a specificity of 83.7% (Figure 2). The Hosmer-Lemeshow test yielded a non-significant result (*χ*^2^ = 5.967, *P* = 0.651), indicating no statistical difference between the predicted and observed frequencies. The calibration curve further confirmed the high consistency between the nomogram's predictions and actual outcomes (Figure 3). Internal validation using 1,000 bootstrap resamples showed minimal bias, confirming the model's robustness.

**Figure 1:**
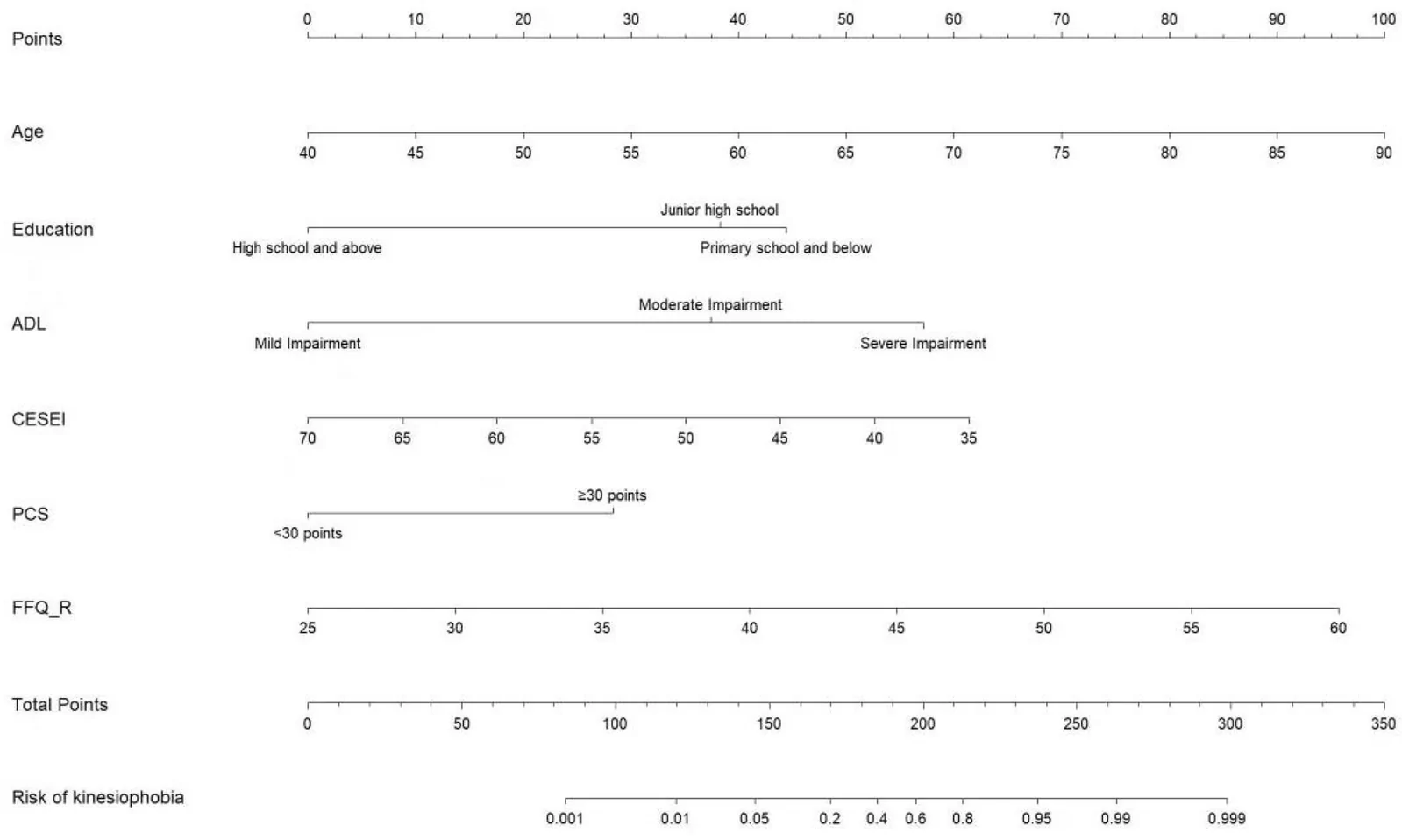
Nomogram model for predicting kinesiophobia in post-CABG patients.

**Figure 2:**
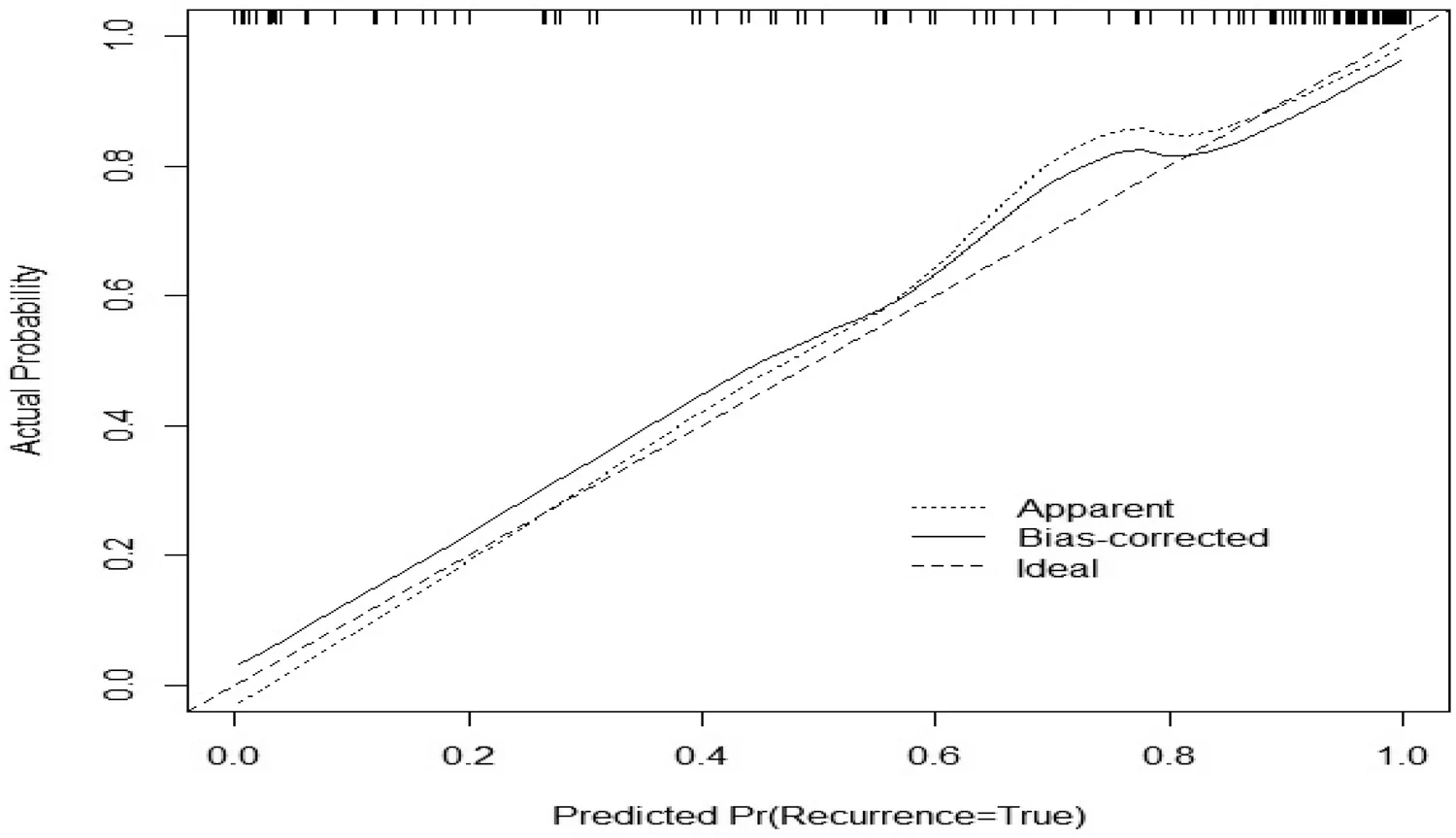
Calibration curve of the nomogram model for predicting kinesiophobia in post-CABG patients.

**Figure 3:**
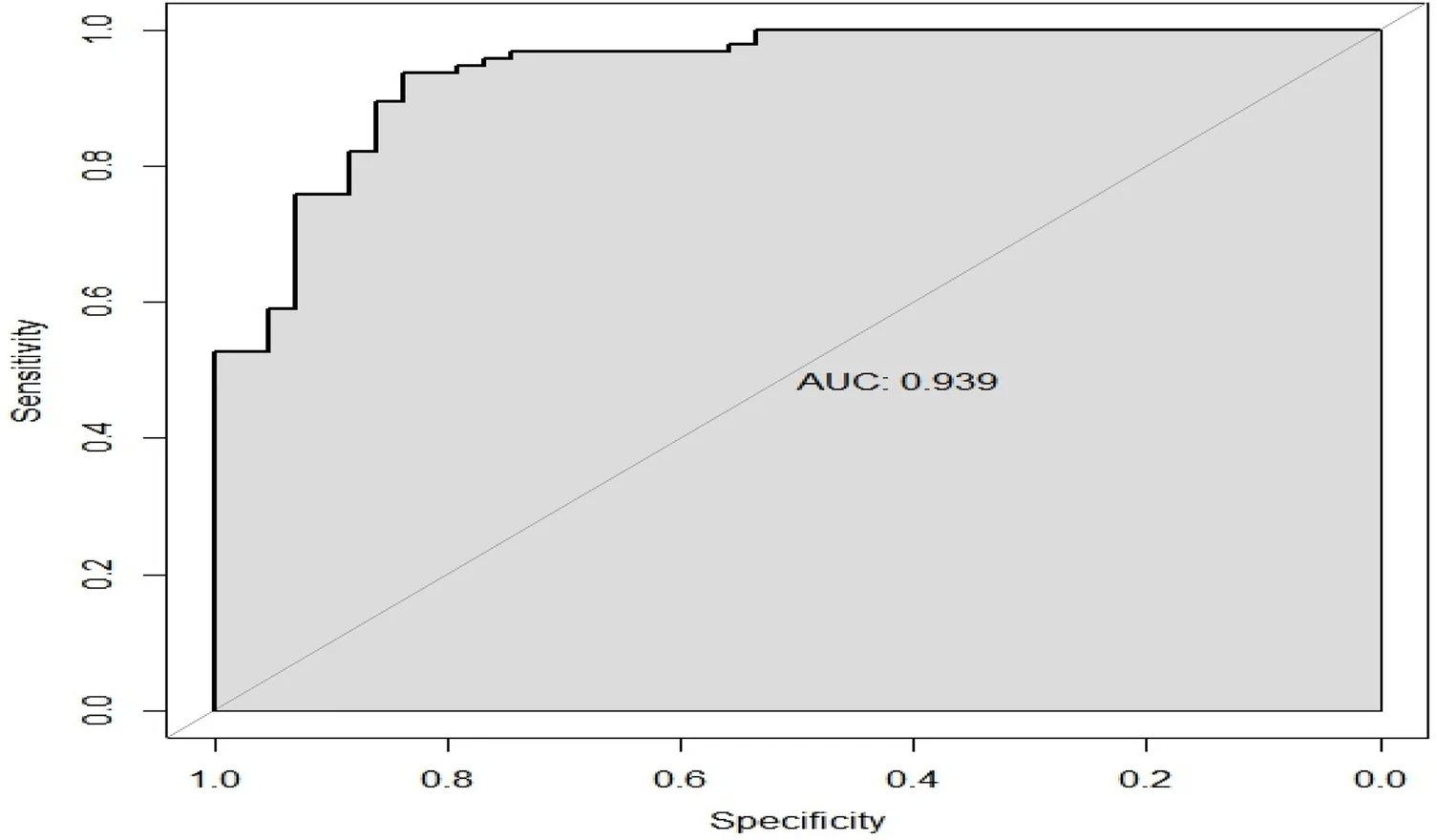
ROC curve of the nomogram model for predicting kinesiophobia in post-CABG patients.

## 4 Discussion

This study confirmed that the incidence of kinesiophobia in patients after CABG was 68.84%. This finding is consistent with previous research, which has reported a high prevalence of kinesiophobia in this specific patient population, with rates ranging from 65.69% to 77.91% (13). This substantial rate underscores kinesiophobia as a significant clinical issue that can severely compromise patient recovery following CABG surgery. The high incidence of kinesiophobia has profound negative implications for patient prognosis. It is known to prolong hospital discharge times, reduce quality of life, and seriously affect recovery outcomes (13). Kinesiophobia directly undermines a patient's adherence to exercise-centered cardiac rehabilitation, which is a safe and effective component of recovery. This lack of engagement can lead to muscle atrophy, diminished cardiovascular health, and increased economic burdens for both families and society.

Our multivariate analysis identified several independent predictors, offering profound insights into the profile of the at-risk patient. First, advanced age emerged as a significant demographic risk factor. The mechanism behind this is multifaceted; older patients not only face age-related physiological declines, such as diminished muscle strength and balance, but they also frequently present with multiple comorbidities, which can heighten their perception of risks associated with postoperative activity (14, 15). As related research indicates, the state of frailty, which is common among elderly cardiac surgery patients, is closely linked with both kinesiophobia and a fear of falling, creating a vicious cycle (16). Second, a lower educational level was confirmed as another key risk factor (7, 17). This extends beyond mere access to information and touches upon the core role of health literacy in patient recovery. Individuals with lower health literacy may face greater challenges in comprehending complex medical information, evaluating activity-related risks, and effectively implementing self-managed rehabilitation plans, thereby making them more susceptible to unnecessary fear and catastrophic thinking. While pain education effectively reduces pain and kinesiophobia (18), the early implementation of targeted preventive measures is crucial for preventing the onset of kinesiophobia. In this context, the “Keep Your Move in the Tube” (KYMITT) strategy offers a practical, patient-centered approach that encourages patients to keep their arms close to the body during daily activities, shortening the lever arm and lessening sternal stress, while replacing traditional restrictive precautions with a pain-guided, movement-based model that promotes functional independence and directly counteracts fear-avoidance behaviors (19).

On a functional and psychosocial level, our findings are strongly corroborated by recent literature (20, 21). A higher degree of ADL disability was a powerful predictor. In the early postoperative period, a patient's dependence on others for basic daily activities like dressing, eating, and walking can directly erode their sense of self and their feeling of control over their own body, leading them to internalize the identity of a “fragile patient” and thus resist any physically demanding activity. This functional dependence is often intricately linked with another significant predictor we identified: a fear of falling (21). As recent studies have detailed, a complex interplay exists among functional dependence, fear of falling, and kinesiophobia in postoperative older adults, forming a negative feedback loop that is difficult to break (21, 22).

Furthermore, our model underscores the central role of psychological factors. Lower cardiac rehabilitation self-efficacy was identified as a absence of a protective factor (7, 23). Self-efficacy, a cornerstone of Bandura's social cognitive theory, is a critical internal driver for health behavior change. Patients who lack confidence in their ability to engage in rehabilitation activities safely are more likely to retreat and avoid them when faced with discomfort or challenges. Conversely, a higher degree of pain catastrophizing acted as a potent risk driver. Pain catastrophizing is more than a simple amplification of pain (23), and it involves a series of negative cognitive processes, such as rumination, magnification of threat, and feelings of helplessness. This mindset aligns perfectly with the theoretical framework of the fear-avoidance model, where catastrophic thinking leads directly to a fear of activity, which in turn triggers avoidance behaviors, ultimately resulting in functional decline and poorer long-term prognoses (24).

The primary clinical contribution of this research lies in the development and validation of a practical predictive nomogram. In the landscape of clinical decision support tools, nomograms are favored for their intuitive and user-friendly nature, and they have been successfully applied in predicting postoperative complications, such as delirium, in the field of cardiac surgery (25). Our model demonstrated excellent discrimination (AUC = 0.939) and good calibration, positioning it as a reliable aid for clinical decision-making. In the early postoperative phase, clinicians can input a few simple variables to rapidly quantify a patient's risk of developing kinesiophobia. This risk stratification allows for a more targeted allocation of healthcare resources: high-risk patients could be preemptively referred to clinical psychologists for cognitive-behavioral therapy or assigned to senior physical therapists for one-on-one, confidence-building rehabilitation sessions. Such proactive measures can effectively intervene before fear-avoidance behaviors become entrenched.

Nevertheless, several limitations should be acknowledged when interpreting our findings. First, although all scales used in this study have been translated into Chinese and their psychometric properties have been validated in local populations, subtle cross-cultural differences in item interpretation cannot be entirely excluded, and further validation studies specifically targeting post-CABG patients are warranted. Second, the nomogram was derived and internally validated in a single-center sample; its high AUC (>0.90) requires external validation in independent, multicenter cohorts before clinical implementation. Third, the current predictors rely on multiple questionnaire-based assessments, which may limit the nomogram's bedside utility. Future efforts should focus on simplifying the prediction model to include only easily accessible clinical variables (e.g., demographic and routine medical data) or ultra-short questionnaire versions without compromising predictive performance.

Despite these caveats, our findings provide valuable insights into the psychosocial determinants of kinesiophobia after CABG and lay a foundation for developing targeted, multidisciplinary interventions to reduce fear-avoidance behaviors and promote active participation in cardiac rehabilitation, ultimately improving patient outcomes. Future research with larger, prospective, multicenter designs and simplified assessment tools is warranted.

## 5 Conclusions

In conclusion, kinesiophobia is a highly prevalent issue among patients recovering from CABG surgery. This study identified advanced age, lower educational level, ADL disability, low cardiac exercise self-efficacy, pain catastrophizing, and fear of falling as significant, independent predictors. The novel nomogram developed from these factors demonstrates excellent predictive accuracy and calibration. This tool offers clinicians a valuable method for the early identification of high-risk individuals, paving the way for targeted, multidisciplinary interventions aimed at reducing fear-avoidance behaviors and promoting active participation in cardiac rehabilitation, ultimately leading to improved patient outcomes.

## Data Availability

The original contributions presented in the study are included in the article/Supplementary Material, further inquiries can be directed to the corresponding author.
