# Supplementary material for: Analysis of risk factors for kinesiophobia after coronary artery bypass grafting and construction of a nomogram prediction model
Source: Front Cardiovasc Med. 2026 Oct 26;13:1742685. doi: 10.3389/fcvm.2026.1742685 (PMC13745263; doi:10.3389/fcvm.2026.1742685)
Supplement: Supplementary file 1 [file Table1.docx]

Stab. 1 Assignment of Independent Variables for Kinesiophobia in Post-CABG Patients

| Variables | Assignment method |
| --- | --- |
| Age | Original value |
| Sex | Male=1, Female=2 |
| Education Level | Primary school and below=1, Junior high school=2,  High school and above=3 |
| Caregiver | Children=1, Spouse=2, Caregiver and others=3 |
| Comorbidities | Original value |
| Number of Grafts | ≤2=1，>2=2 |
| Ventilator Use Duration | Original value |
